# Supplementary material for: SmartHisto: Bayesian active learning for histology images
Source: PLoS Comput Biol. 2026 Sep 17;22(9):e1013611. doi: 10.1371/journal.pcbi.1013611 (PMC13600622; doi:10.1371/journal.pcbi.1013611)
Supplement: Supplementary file 1 [file pcbi.1013611.s001.pdf]

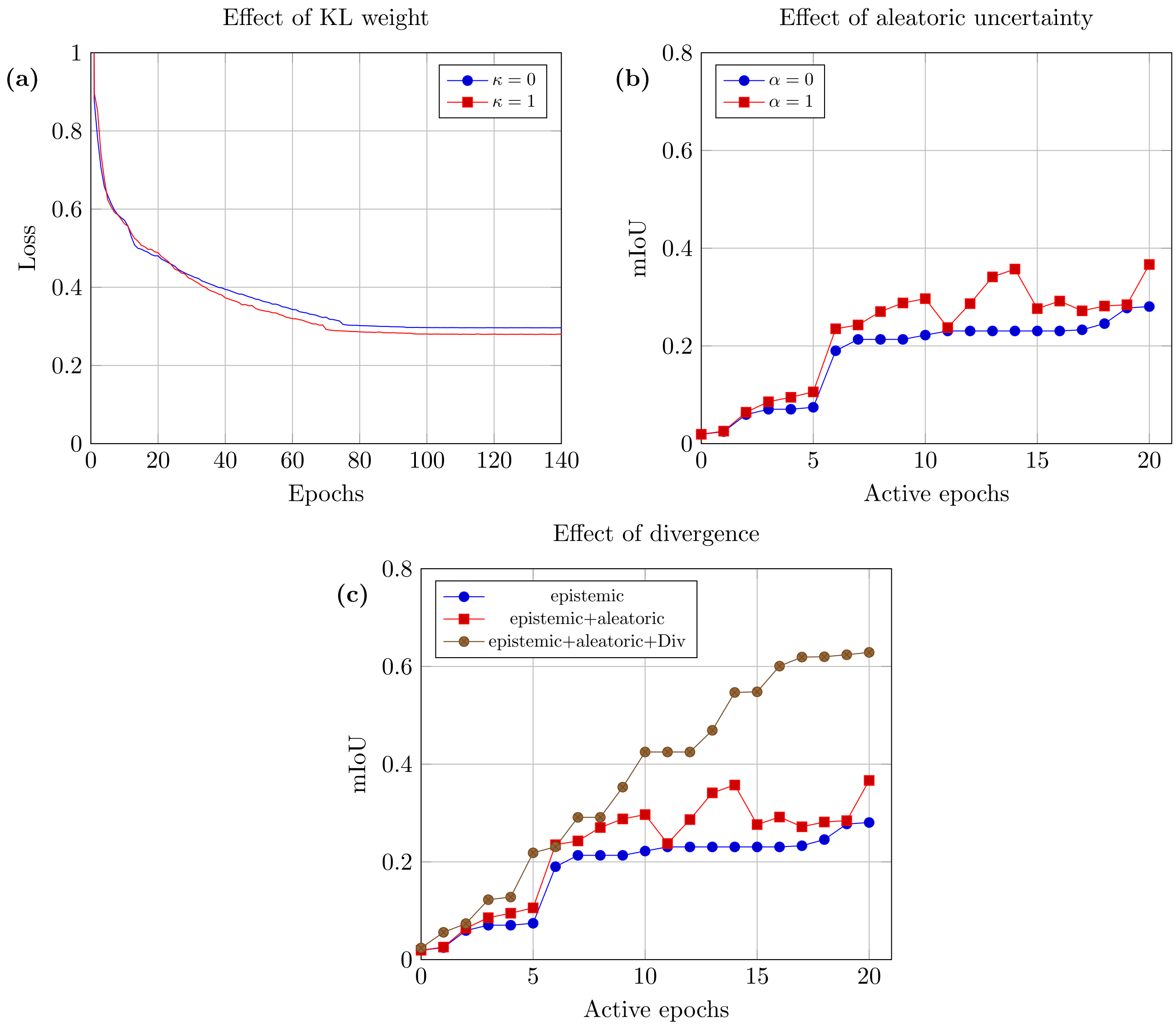
Effect of KL weight
(a)
Loss
Epochs
$\kappa = 0$
$\kappa = 1$
Effect of aleatoric uncertainty
(b)
mIoU
Active epochs
$\alpha = 0$
$\alpha = 1$
Effect of divergence
(c)
mIoU
Active epochs
epistemic
epistemic+aleatoric
epistemic+aleatoric+Div
