# Supplementary material for: SmartHisto: Bayesian active learning for histology images
Source: PLoS Comput Biol. 2026 Sep 17;22(9):e1013611. doi: 10.1371/journal.pcbi.1013611 (PMC13600622; doi:10.1371/journal.pcbi.1013611)
Supplement: Supplementary file 2 [file pcbi.1013611.s002.pdf]

1 Reviewer 1

- Some practical details about the annotation workflow are missing, for example, the real time savings for human experts.
  We have revised the manuscript to include additional discussion to indicate where we believe this approach to be useful, and we provide additional directions for research where we believe SmartHisto could be refined. The proposed method serves as a general framework on how large-scale segmentation models may be constructed in situations with scarce annotation data.

- The discussion of limitations is quite brief. There are few comments on situations where the method could have problems, such as scaling to larger datasets or dealing with more complex annotation tasks.
  We have specifically addressed this with a new paragraph in the discussion section. We note that SmartHisto has limitations because the segmented classes are mutually exclusive; we used relatively consistent magnification for training; and that we only explored the method using a single histopathological stain. All these areas represent interesting future directions for research that could add value to the approach and use in the field.

- More explanations about how clinical users can benefit from this pipeline would help readers from medical backgrounds.
  We have amended the discussion section to address how the proposed approach can be implemented as a cost-effective solution for large-scale image annotation.

2 Reviewer 2

- Bayesian Neural Network Architecture - The weights and biases of the Bayesian Neural Network (BNN) are defined using Gaussian noise ϵ where $\epsilon \sim N(1,0)$. A normal distribution with mean 1 and variance 0 is deterministic, meaning the value is fixed at 1. Please verify this notation, as standard practice for BNN weight sampling typically involves noise centered around zero, e.g., $N(0,1)$ or $N(0,\sigma^2)$.
  Thank you. This was a typograpical error and has been corrected. The noise added is sampled from a Gaussian distribution $N(0,1)$, following the standard approach described in the reviewer comment, and not $N(1,0)$.

- Scaling factors (κ and α): The ablation study identified that a scaling factor of $\kappa = 1$ for the KL divergence term in the loss function and a scaling factor of $\alpha = 1$ for the aleatoric uncertainty weight provided the best overall performance. Since these factors critically balance the model's complexity (KL divergence) and the influence of irreducible noise (aleatoric uncertainty) in sample selection, do the authors consider the

chosen values are consistently the optimal choice for distinct datasets and segmentation tasks or might it require dataset-specific tuning?
These parameters, derived from our empirical analysis of the pathology dataset, serve as a reasonable starting point for many datasets. There may be datasets where these parameters are not ideal and require tuning, but these parameters are likely to converge after more active epochs. We include an explanation for these parameters in the 'Selecting Regions for Annotation' section of the manuscript.

- Superpixeling parameters: The methodology relies heavily on superpixeling using SLIC to cluster uncertain pixels into regions for annotation. The effectiveness of the divergence-based uncertainty metric depends directly on the quality and size of these superpixels. For reproducibility and to assess how the grouping impacts the identification of fine histopathological structures, please detail the specific parameters used for the SLIC implementation (e.g., compactness, desired number of superpixels).
  We used the default parameters provided by the SLIC. We have detailed the exact parameter values in revised manuscript: 1000 segments with a compactness of 28 performed well for all the tested datasets.

- Prediction Probability Threshold - In the Experimental Settings, the authors state that pixels with computed probabilities of 0.6 or more were considered to belong to the respective output class. Please provide justification for selecting this non-standard threshold of 0.6, or confirm if this value was determined empirically to maximize performance.
  We amend the manuscript to address this comment. The threshold of 0.6 was empirically selected as a conservative cutoff. This value can be changed for other datasets if needed.

- GlaS Dataset - It is stated that the original pixel resolution of the GlaS dataset images is 0.465µm, and all images were registered to 256x256 pixels for training. Given that these images originate from Whole-Slide Images (WSIs) and their initial sizes varied, please specify the typical or maximum dimensions of the 167 original images used from the GlaS dataset before they were uniformly registered to 256x256, for a clearer understanding of the data scale.
  These details have been added to the revised manuscript. Each image was originally of dimension 775×552.

- Data augmentation - The authors specifically exclude color channel shifts and swaps from the image augmentation strategy, arguing that such alterations might hinder the impact of histopathology tissue staining and model performance. However, it is indicated that both the GlaS dataset exhibits high inter-subject variability in stain distribution and the Pulmonary dataset contains variations due to differences in the staining practices and image scanners. How these differences caused by the stain processes impact in the generalization capability of the models?

We have amended this section of the manuscript to improve clarity. We made the decision to exclude color channel shifts as they may obscure the variability seen in staining that is described by the reviewer. The stain variability in image training from the GLaS and Pulmonary datasets is captured as informative areas for the model. This has an outcome of generating a more robust and generalizable model.

- Figure 4 - Part of the description in the caption does not seem to correspond to the figure.
  Thank you. This is figure is now labeled Fig 5, and the caption has been corrected.

- Computational Efficiency - The authors claim that SmartHisto achieves superior models without compromising memory efficiency. Since BNNs replace fixed weight parameters with distributions, which often introduce computational or memory overhead compared to classical point-estimate models, please provide concrete metrics to support this claim. Specifically, including a direct comparison of training time and/or memory usage between the Bayesian-UNet used by SmartHisto and the SOTA point-estimate models (QBC, DEAL) would validate the efficiency statement.
  We have clarified our experiments and included a new table to the manuscript (Table 1). This table includes the performance, memory requirements, and runtime of each method. This supports our contention that SmartHisto achieves a superior model without compromising memory efficiency.

3 Reviewer 3

3.1 major points

- generally, the discussion is greatly lacking and requires a meaningful interpretation of the model's strengths and drawbacks, and an explanation for why true SOTA active learning methods were not benchmarked (e.g. deep ensembles, Variational Adversarial Active Learning etc)
  We have amended our discussion on current limitations and future research directions. We do not compete SmartHisto against all methods, including Deep ensembles and Variational Adversarial Active learning as the reviewer suggests, because these do not provide pixel level uncertainty estimates and instead provide only whole image uncertainty estimates. This difference makes it hard to compare as they are fundamentally different and have different outcomes. We include these alternate approaches in the manuscript, and briefly state why they were not tested.

- From Fig 2, SMART only outperforms other methods after 80 active epochs. To me this doesn't seem to be a very large improvement, please address this in the discussion.
  Thank you, we have provided a potential explanation for this behavior

which was more apparent with the GLaS dataset in the revised discussion. Specifically, the GLaS dataset includes portions of glass side that were not in the Pumonary dataset. SmartHisto achieves a higher mIoU at the end of active training, and we believe it has utility.

- in digital pathology, workflows typically involve region selection decision within entire WSI, not on pre-selected tiles. How does your model perform on a dataset with entire WSIs? how would you use your model to selected regions for annotations practically in this kind of setting?
  The full pulmonary dataset includes a full WSI and SmartHisto samples tiles based on uncertainty. In the case of multiple WSI images, the runtime to annotate the full image will scale with the number of WSI images. This detail has been added to the discussion section.

- Bayesian learning is notoriously slow, please report the speed of every method your benchmarked and a discussion of this limitation.
  We have revised the manuscript to include a new table (Table 1). This includes the improved performance, memory requirements and runtime of SmartHisto relative to the other tested methods.

- Fig 2: as the values are very close together, so it's hard to see how much of an improvement there is from other methods. Adding a table would help the reader understand the amount of improvement.
  A table (Table 1) has been added to the manuscript to indicate the improved performance, memory requirements and runtime of each method.

- it would be a lot more helpful to see the performance vs the % sample used as opposed to the number of active learning epochs (Fig2 and 3). Can you provide a version of these figures with % training samples as the x-axis?
  Figures 2 and 4 (formerly 2 and 3, respectively) have been modified to include the % dataset being used at each active epoch for training.

- why were larger datasets not used as benchmarks (e.g., TIGER)?
  We appreciate the suggestion, but upon extensive searching we are unable to find a TIGER dataset.

- the code repository is inaccessible
  We have verified that the git repo is accessible and public.

- there is no link to the pulmonary dataset, which is required by the journal. Please make it available.
  The dataset has now been uploaded to Zenodo with a DOI of 10.5281/zenodo.18421739.

3.2 minor points

- please show examples of your input images as-is (not crops) from both the GlaS and your pulmonary dataset to show the reader what they look like.

We include a new figure (Fig 3) that includes input images without crops.

- Fig2: the first part of the curves is hidden by the legend, please fix this.
  We have corrected the legend to show the underlying curves.
- please expand all acronyms on first mention (e.g., ENT, RAND etc)
  The text has been corrected to include all the expanded acronyms.
- Fig 5 and 6: the column labels are too small and need to be bigger
  We have amended both Fig 5 and 6 to include larger column labels; each column is also described in the figure legend.
