# Supplementary material for: SmartHisto: Bayesian active learning for histology images
Source: PLoS Comput Biol. 2026 Sep 17;22(9):e1013611. doi: 10.1371/journal.pcbi.1013611 (PMC13600622; doi:10.1371/journal.pcbi.1013611)
Supplement: Supplementary file 3 [file pcbi.1013611.s003.pdf]

# Response to Reviewers

sriram

## Reviewer #3

**Concern:** *"In digital pathology, workflows typically involve region selection decision within entire WSIs, not on pre-selected tiles. How does your model perform on a dataset with entire WSIs? How would you use your model to select regions for annotation practically in this kind of setting? ... Basically, picture the reader as someone interested in using SmartHisto. This reader has a WSI to annotate, how much less work would it take to annotate that WSI if they incorporate SmartHisto?"*

**Response:** We have added a new analysis to the revised manuscript in Table 9 of Performance on the TIGER ROI Dataset. The TIGER dataset consists of 153 ROI tiles and we report the number of tiles that must be annotated before each method reaches a target segmentation quality of mIoU.

A pathologist using SmartHisto needs to annotate only 43% of the available tiles to reach a practical segmentation quality, compared with 49% for random selection and 53% for entropy sampling. Note that some baselines never reach this threshold at all. Furthermore, SmartHisto's peak mIoU of 0.4181 is not matched by any baseline regardless of how many tiles they annotate, confirming that the benefit is not merely speed but also final model quality.

**Concern:** *"Why were larger datasets not used as benchmarks (e.g., TIGER)? The TIGER dataset is a well-publicised segmentation challenge ... the question still stands: why were larger datasets not used as benchmarks?"*

**Response:** The full experimental setup, results, and analysis are reported in the new subsection TIGER ROI Dataset and Performance on the TIGER ROI Dataset. Key findings are:

- SmartHisto achieves the highest peak mIoU of 0.4181 across all 13 active learning iterations.
- RAND and QBC follow at 0.3964 and 0.3859, respectively; ENT and MAR trail at 0.3754 and 0.3438.
- SmartHisto reaches competitive performance (>0.38 mIoU) earlier in the active learning curve than any baseline, demonstrating superior sample

efficiency on a large, class-imbalanced dataset.

- Qualitative predictions on the highest-scoring validation tiles are shown in Figures 7 and 8 of the revised manuscript.
