# Supplementary material for: SmartHisto: Bayesian active learning for histology images
Source: PLoS Comput Biol. 2026 Sep 17;22(9):e1013611. doi: 10.1371/journal.pcbi.1013611 (PMC13600622; doi:10.1371/journal.pcbi.1013611)
Supplement: Peer review history

# Peer review: Decision Letter 0

**DOI:** 10.1371/journal.pcbi.1013611.r001

**Authors:** Virginia E. Pitzer, Guillermo Lorenzo

1 Dec 2025

SmartHisto: Bayesian Active Learning for Histology Images

PLOS Computational Biology

Dear Dr. Vijendran,

Thank you for submitting your manuscript to PLOS Computational Biology. After careful consideration, we feel that it has merit but does not fully meet PLOS Computational Biology's publication criteria as it currently stands. Therefore, we invite you to submit a revised version of the manuscript that addresses the points raised during the review process.

Please submit your revised manuscript by Jan 31 2026 11:59PM. If you will need more time than this to complete your revisions, please reply to this message or contact the journal office at ploscompbiol@plos.org. When you're ready to submit your revision, log on to https://www.editorialmanager.com/pcompbiol/ and select the 'Submissions Needing Revision' folder to locate your manuscript file.

Please include the following items when submitting your revised manuscript:

* A rebuttal letter that responds to each point raised by the editor and reviewer(s). You should upload this letter as a separate file labeled 'Response to Reviewers'. This file does not need to include responses to formatting updates and technical items listed in the 'Journal Requirements' section below.

* A marked-up copy of your manuscript that highlights changes made to the original version. You should upload this as a separate file labeled 'Revised Manuscript with Track Changes'.

* An unmarked version of your revised paper without tracked changes. You should upload this as a separate file labeled 'Manuscript'.

If you would like to make changes to your financial disclosure, competing interests statement, or data availability statement, please make these updates within the submission form at the time of resubmission. Guidelines for resubmitting your figure files are available below the reviewer comments at the end of this letter

We look forward to receiving your revised manuscript.

Kind regards,

Guillermo Lorenzo

Academic Editor

PLOS Computational Biology

Virginia Pitzer

Editor-in-Chief

PLOS Computational Biology

**Additional Editor Comments (if provided):**

**Journal Requirements:**

If the reviewer comments include a recommendation to cite specific previously published works, please review and evaluate these publications to determine whether they are relevant and should be cited. There is no requirement to cite these works unless the editor has indicated otherwise.

[Note: HTML markup is below. Please do not edit.]

**Reviewers' comments:**

Reviewer's Responses to Questions

**Comments to the Authors:**

**Please note here if the review is uploaded as an attachment.**

Reviewer #1: This paper presents SmarHtisto, a new method for active learning applied to histology image segmentation. The main idea is to reduce the annotation efforts by using Bayesian NN to estimate uncertainty, and select the regions that are more informative for annotation. The authors validate their approach on several benchmark datasets and demonstrate better results compared to current methods.

Points to improve:

- Some practical details about the annotation workflow are missing, for example, the real time savings for human experts.

- The discussion of limitations is quite brief. There are few comments on situations where the method could have problems, such as scaling to larger datasets or dealing with more complex annotation tasks.

- More explanations about how clinical users can benefit from this pipeline would help readers from medical backgrounds.

Despite the above points, the manuscript offers relevant technical advances and clear competitive results. The experimental validation is robust and the implementation details are mostly sufficient. The selection of references is well balanced and includes the most important previous work. The manuscript will benefit from minor improvements in the discussion and practical recommendations, but it already provides significant contribution to the field. Therefore, I recommend acceptance after minor revisions.

Reviewer #2: SmartHisto is introduced as a novel Active Learning sample selection heuristic designed specifically for the semantic segmentation of histology Whole-Slide Images. This methodology addresses critical challenges in histopathology, such as the high costs associated with image annotation and the limited use of archived material, which typically constrain the effectiveness of traditional machine learning pipelines. It relies on a Bayesian Neural Network to estimate aleatoric and epistemic uncertainty, and uses superpixeling to group pixels into meaningful regions based on these uncertainty measures. A new divergence-based metric is then applied to rank samples, prioritizing those expected to provide the most information. Experimental results show that SmartHisto outperforms traditional and state-of-the-art sampling strategies while requiring fewer annotated samples.

Overall, the study presents a promising and well-structured pipeline with potential to improve segmentation performance in low-annotation settings. However, several details regarding the methodology and evaluation remain unclear and should be clarified to ensure the reproducibility of the study and to allow a proper assessment of the robustness and generalizability of the approach.

- Bayesian Neural Network Architecture - The weights and biases of the Bayesian Neural Network (BNN) are defined using Gaussian noise ϵ where ϵ∼N(1,0). A normal distribution with mean 1 and variance 0 is deterministic, meaning the value is fixed at 1. Please verify this notation, as standard practice for BNN weight sampling typically involves noise centered around zero, e.g., N(0,1) or N(0,σ^2).

- Scaling factors (κ and α): The ablation study identified that a scaling factor of κ=1 for the KL divergence term in the loss function and a scaling factor of α=1 for the aleatoric uncertainty weight provided the best overall performance. Since these factors critically balance the model's complexity (KL divergence) and the influence of irreducible noise (aleatoric uncertainty) in sample selection, do the authors consider the chosen values are consistently the optimal choice for distinct datasets and segmentation tasks or might it require dataset-specific tuning?

- Superpixeling parameters: The methodology relies heavily on superpixeling using SLIC to cluster uncertain pixels into regions for annotation. The effectiveness of the divergence-based uncertainty metric depends directly on the quality and size of these superpixels. For reproducibility and to assess how the grouping impacts the identification of fine histopathological structures, please detail the specific parameters used for the SLIC implementation (e.g., compactness, desired number of superpixels).

- Prediction Probability Threshold - In the Experimental Settings, the authors state that pixels with computed probabilities of 0.6 or more were considered to belong to the respective output class. Please provide justification for selecting this non-standard threshold of 0.6, or confirm if this value was determined empirically to maximize performance.

-GlaS Dataset - It is stated that the original pixel resolution of the GlaS dataset images is 0.465µm, and all images were registered to 256x256 pixels for training. Given that these images originate from Whole-Slide Images (WSIs) and their initial sizes varied, please specify the typical or maximum dimensions of the 167 original images used from the GlaS dataset before they were uniformly registered to 256x256, for a clearer understanding of the data scale.

- Data augmentation - The authors specifically exclude color channel shifts and swaps from the image augmentation strategy, arguing that such alterations might hinder the impact of histopathology tissue staining and model performance. However, it is indicated that both the GlaS dataset exhibits high inter-subject variability in stain distribution and the Pulmonary dataset contains variations due to differences in the staining practices and image scanners. How these differences caused by the stain processes impact in the generalization capability of the models?

- Figure 4 - Part of the description in the caption does not seem to correspond to the figure.

- Computational Efficiency - The authors claim that SmartHisto achieves superior models without compromising memory efficiency. Since BNNs replace fixed weight parameters with distributions, which often introduce computational or memory overhead compared to classical point-estimate models, please provide concrete metrics to support this claim. Specifically, including a direct comparison of training time and/or memory usage between the Bayesian-UNet used by SmartHisto and the SOTA point-estimate models (QBC, DEAL) would validate the efficiency statement.

Reviewer #3: major points

- generally, the discussion is greatly lacking and requires a meaningful interpretation of the model's strengths and drawbacks, and an explanation for why true SOTA active learning methods were not benchmarked (e.g. deep ensembles, Variational Adversarial Active Learning etc)

- From Fig 2, SMART only outperforms other methods after 80 active epochs. To me this doesn't seem to be a very large improvement, please address this in the discussion.

- in digital pathology, workflows typically involve region selection decision within entire WSI, not on pre-selected tiles. How does your model perform on a dataset with entire WSIs? how would you use your model to selected regions for annotations practically in this kind of setting?

- Bayesian learning is notoriously slow, please report the speed of every method your benchmarked and a discussion of this limitation.

- Fig 2: as the values are very close together, so it's hard to see how much of an improvement there is from other methods. Adding a table would help the reader understand the amount of improvement.

- it would be a lot more helpful to see the performance vs the % sample used as opposed to the number of active learning epochs (Fig2 and 3). Can you provide a version of these figures with % training samples as the x-axis?

- why were larger datasets not used as benchmarks (e.g., TIGER)?

- the code repository is inaccessible

- there is no link to the pulmonary dataset, which is required by the journal. Please make it available.

minor points

- please show examples of your input images as-is (not crops) from both the GlaS and your pulmonary dataset to show the reader what they look like.

- Fig2: the first part of the curves is hidden by the legend, please fix this.

- please expand all acronyms on first mention (e.g., ENT, RAND etc)

- Fig 5 and 6: the column labels are too small and need to be bigger

**********

**Have the authors made all data and (if applicable) computational code underlying the findings in their manuscript fully available?**

The PLOS Data policy requires authors to make all data and code underlying the findings described in their manuscript fully available without restriction, with rare exception (please refer to the Data Availability Statement in the manuscript PDF file). The data and code should be provided as part of the manuscript or its supporting information, or deposited to a public repository. For example, in addition to summary statistics, the data points behind means, medians and variance measures should be available. If there are restrictions on publicly sharing data or code —e.g. participant privacy or use of data from a third party—those must be specified.

Reviewer #1: Yes

Reviewer #2: Yes

Reviewer #3: **No:**  they have not made their Pulmonary Histology Dataset available and provide no statement about this.

**********

PLOS authors have the option to publish the peer review history of their article (what does this mean?). If published, this will include your full peer review and any attached files.

If you choose “no”, your identity will remain anonymous but your review may still be made public.

**Do you want your identity to be public for this peer review?**  For information about this choice, including consent withdrawal, please see our Privacy Policy.

Reviewer #1: No

Reviewer #2: No

Reviewer #3: No

[NOTE: If reviewer comments were submitted as an attachment file, they will be attached to this email and accessible via the submission site. Please log into your account, locate the manuscript record, and check for the action link "View Attachments". If this link does not appear, there are no attachment files.]

**Figure resubmission:**

**Reproducibility:**

?>

# Author comment: Author response to Decision Letter 1

**DOI:** 10.1371/journal.pcbi.1013611.r002

13 Feb 2026

Attachment Submitted filename: Response to Reviewers.pdf

# Peer review: Decision Letter 1

**DOI:** 10.1371/journal.pcbi.1013611.r003

**Authors:** Virginia E. Pitzer, Guillermo Lorenzo

5 Apr 2026

PCOMPBIOL-D-25-02064R1

SmartHisto: Bayesian Active Learning for Histology Images

PLOS Computational Biology

Dear Dr. Vijendran,

Thank you for submitting your manuscript to PLOS Computational Biology. After careful consideration, we feel that it has merit but does not fully meet PLOS Computational Biology's publication criteria as it currently stands. Therefore, we invite you to submit a revised version of the manuscript that addresses the points raised during the review process.

Please submit your revised manuscript by Jun 05 2026 11:59PM. If you will need more time than this to complete your revisions, please reply to this message or contact the journal office at ploscompbiol@plos.org. When you're ready to submit your revision, log on to https://www.editorialmanager.com/pcompbiol/ and select the 'Submissions Needing Revision' folder to locate your manuscript file.

Please include the following items when submitting your revised manuscript:

* A letter that responds to each point raised by the editor and reviewer(s). You should upload this letter as a separate file labeled 'Response to Reviewers'. This file does not need to include responses to formatting updates and technical items listed in the 'Journal Requirements' section below.

* A marked-up copy of your manuscript that highlights changes made to the original version. You should upload this as a separate file labeled 'Revised Manuscript with Track Changes'.

* An unmarked version of your revised paper without tracked changes. You should upload this as a separate file labeled 'Manuscript'.

If you would like to make changes to your financial disclosure, competing interests statement, or data availability statement, please make these updates within the submission form at the time of resubmission. Guidelines for resubmitting your figure files are available below the reviewer comments at the end of this letter.

We look forward to receiving your revised manuscript.

Kind regards,

Guillermo Lorenzo

Academic Editor

PLOS Computational Biology

Virginia Pitzer

Editor-in-Chief

PLOS Computational Biology

**Additional Editor Comments:**

Dear authors,

I would like to congratulate you in the work carried out to address the concerns raised by the reviewers. While they all agree that the manuscript has improved notably and two of them recommend publication, it seems that two concerns from the thrid reviewer remain unresolved. I strongly reccomend reading the rationale of the two issues raised by this reviewer and revising your manuscript accordingly.

Sincerely,

GL

**Journal Requirements:**

If the reviewer comments include a recommendation to cite specific previously published works, please review and evaluate these publications to determine whether they are relevant and should be cited. There is no requirement to cite these works unless the editor has indicated otherwise.

1) We note that your SmartHisto.pdf, and Manuscript.pdf files are duplicated on your submission. Please remove any unnecessary or old files from your revision, and make sure that only those relevant to the current version of the manuscript are included.

2) Please amend your detailed Financial Disclosure statement. This is published with the article. It must therefore be completed in full sentences and contain the exact wording you wish to be published.

1) State the initials, alongside each funding source, of each author to receive each grant. For example: "This work was supported by the National Institutes of Health (####### to AM; ###### to CJ) and the National Science Foundation (###### to AM).".

If you did not receive any funding for this study, please simply state: u201cThe authors received no specific funding for this work.u201d

3)  Please ensure that the funders and grant numbers match between the Financial Disclosure field and the Funding Information tab in your submission form. Note that the funders must be provided in the same order in both places as well.

**Reviewers' comments:**

Reviewer's Responses to Questions

**Comments to the Authors:**

**Please note here if the review is uploaded as an attachment.**

Reviewer #1: I have carefully reviewed the revised version of the manuscript. All the comments and concerns I raised in the previous review round have been adequately addressed. The authors have satisfactorily clarified the issues and improved the manuscript accordingly.

Therefore, I recommend the manuscript for acceptance in its current form.

Reviewer #2: The authors have carefully addressed the comments raised by the reviewers. The revisions satisfactorily resolve the concerns, and the manuscript has been improved accordingly. I have no further comments.

Reviewer #3: 1) This comment was not sufficiently addressed:

"in digital pathology, workflows typically involve region selection decision within entire WSI, not on pre-selected tiles. How does your model perform on a dataset with entire WSIs? how would you use your model to selected regions for annotations practically in this kind of setting?"

This is a critical point to address as the authors mention the cost of annotating WSIs as a major motivator for this paper, yet it wasn't sufficiently addressed in the response. In their response they mentioned that the pulmonary dataset has a full WSI in it but don't report the performance on it and the % area that need annotations to reach a reasonable performance on it. Basically, picture the reader as someone interested in using SmartHisto. This reader has a WSI to annotate, how much less work would it take to annotate that WSI if they incorporate SmartHisto?

2) "why were larger datasets not used as benchmarks (e.g., TIGER)?"

The TIGER dataset is a well-publicised segmentation challenge with data: https://tiger.grand-challenge.org/Data/

Regardless, that was just an example out of several, so the question still stands: why were larger datasets not used as benchmarks?

**********

**Have the authors made all data and (if applicable) computational code underlying the findings in their manuscript fully available?**

The PLOS Data policy requires authors to make all data and code underlying the findings described in their manuscript fully available without restriction, with rare exception (please refer to the Data Availability Statement in the manuscript PDF file). The data and code should be provided as part of the manuscript or its supporting information, or deposited to a public repository. For example, in addition to summary statistics, the data points behind means, medians and variance measures should be available. If there are restrictions on publicly sharing data or code —e.g. participant privacy or use of data from a third party—those must be specified.

Reviewer #1: Yes

Reviewer #2: None

Reviewer #3: Yes

**********

PLOS authors have the option to publish the peer review history of their article (what does this mean?). If published, this will include your full peer review and any attached files.

If you choose “no”, your identity will remain anonymous but your review may still be made public.

**Do you want your identity to be public for this peer review?**  For information about this choice, including consent withdrawal, please see our Privacy Policy.

Reviewer #1: **Yes:**  Angel Rio-Alvarez

Reviewer #2: No

Reviewer #3: No

**Figure resubmission:**

After uploading your figures to PLOS’s NAAS tool - https://ngplosjournals.pagemajik.ai/artanalysis, NAAS will process the files provided and display the results in the "Uploaded Files" section of the page as the processing is complete. If the uploaded figures meet our requirements (or NAAS is able to fix the files to meet our requirements), the figure will be marked as "fixed" above. If NAAS is unable to fix the files, a red "failed" label will appear above. When NAAS has confirmed that the figure files meet our requirements, please download the file via the download option, and include these NAAS processed figure files when submitting your revised manuscript.

**Reproducibility:**

To enhance the reproducibility of your results, we recommend that authors of applicable studies deposit laboratory protocols in protocols.io, where a protocol can be assigned its own identifier (DOI) such that it can be cited independently in the future. Additionally, PLOS ONE offers an option to publish peer-reviewed clinical study protocols. Read more information on sharing protocols at https://plos.org/protocols?utm_medium=editorial-email&utm_source=authorletters&utm_campaign=protocols

# Author comment: Author response to Decision Letter 2

**DOI:** 10.1371/journal.pcbi.1013611.r004

22 Jun 2026

Attachment Submitted filename: response-to-reviewers.pdf

# Peer review: Decision Letter 2

**DOI:** 10.1371/journal.pcbi.1013611.r005

**Authors:** Virginia E. Pitzer

24 Aug 2026

Dear Mr Vijendran,

We are pleased to inform you that your manuscript 'SmartHisto: Bayesian Active Learning for Histology Images' has been provisionally accepted for publication in PLOS Computational Biology.

Before your manuscript can be formally accepted you will need to complete some formatting changes, which you will receive in a follow up email. A member of our team will be in touch with a set of requests.

Please note that your manuscript will not be scheduled for publication until you have made the required changes, so a swift response is appreciated.

IMPORTANT: The editorial review process is now complete. PLOS will only permit corrections to spelling, formatting or significant scientific errors from this point onwards. Requests for major changes, or any which affect the scientific understanding of your work, will cause delays to the publication date of your manuscript.

Should you, your institution's press office or the journal office choose to press release your paper, you will automatically be opted out of early publication. We ask that you notify us now if you or your institution is planning to press release the article. All press must be co-ordinated with PLOS.

Thank you again for supporting Open Access publishing; we are looking forward to publishing your work in PLOS Computational Biology.

Best regards,

Virginia E. Pitzer, Sc.D.

Editor-in-Chief

PLOS Computational Biology

Virginia Pitzer

Editor-in-Chief

PLOS Computational Biology

***********************************************************

Reviewer's Responses to Questions

**Comments to the Authors:**

**Please note here if the review is uploaded as an attachment.**

Reviewer #3: The text still doesn't answer how your model would perform on a dataset of whole slide images instead of one that's prepatched. However, thank you for adding the TIGER analysis, I think that will make your proposed method a lot more attractive and understandable to the community.

**********

**Have the authors made all data and (if applicable) computational code underlying the findings in their manuscript fully available?**

The PLOS Data policy requires authors to make all data and code underlying the findings described in their manuscript fully available without restriction, with rare exception (please refer to the Data Availability Statement in the manuscript PDF file). The data and code should be provided as part of the manuscript or its supporting information, or deposited to a public repository. For example, in addition to summary statistics, the data points behind means, medians and variance measures should be available. If there are restrictions on publicly sharing data or code —e.g. participant privacy or use of data from a third party—those must be specified.

Reviewer #3: Yes

**********

PLOS authors have the option to publish the peer review history of their article (what does this mean?). If published, this will include your full peer review and any attached files.

If you choose “no”, your identity will remain anonymous but your review may still be made public.

**Do you want your identity to be public for this peer review?**  For information about this choice, including consent withdrawal, please see our Privacy Policy.

Reviewer #3: No

# Editor report: Acceptance letter

**DOI:** 10.1371/journal.pcbi.1013611.r006

**Authors:** Virginia E. Pitzer

PCOMPBIOL-D-25-02064R2

SmartHisto: Bayesian Active Learning for Histology Images

Dear Dr Vijendran,

I am pleased to inform you that your manuscript has been formally accepted for publication in PLOS Computational Biology. Your manuscript is now with our production department and you will be notified of the publication date in due course.

The corresponding author will soon be receiving a typeset proof for review, to ensure errors have not been introduced during production. Please review the PDF proof of your manuscript carefully, as this is the last chance to correct any errors. Please note that major changes, or those which affect the scientific understanding of the work, will likely cause delays to the publication date of your manuscript.

Soon after your final files are uploaded, unless you have opted out, the early version of your manuscript will be published online. The date of the early version will be your article's publication date. The final article will be published to the same URL, and all versions of the paper will be accessible to readers.

For Research, Software, and Methods articles, you will receive an invoice from PLOS for your publication fee after your manuscript has reached the completed accept phase. If you receive an email requesting payment before acceptance or for any other service, this may be a phishing scheme. Learn how to identify phishing emails and protect your accounts at https://explore.plos.org/phishing.

Thank you again for supporting PLOS Computational Biology and open-access publishing. We are looking forward to publishing your work!

With kind regards,

Janani Seenivasan

PLOS Computational Biology | Carlyle House, Carlyle Road, Cambridge CB4 3DN | United Kingdom ploscompbiol@plos.org | Phone +44 (0) 1223-442824 | ploscompbiol.org | @PLOSCompBiol
